# Role of *ASXL1* mutation in impaired hematopoiesis and cellular senescence

**DOI:** 10.18632/oncotarget.26423

**Published:** 2018-12-07

**Authors:** Masahiro Uni, Mineo Kurokawa

**Affiliations:** Department of Hematology and Oncology, Graduate School of Medicine, The University of Tokyo, Hongo, Bunkyō, Tokyo, Japan

**Keywords:** ASXL1, cellular senescence, low-risk MDS

Progress in the field of next-generation sequencing has identified variety of previously unknown recurrent somatic mutations in a wide spectrum of hematological disorders. Among them, mutations in histone modifier additional sex combs like 1 (*ASXL1*) have been identified as a driver mutation not only in myeloid malignancies, but also in healthy individuals with clonal hematopoiesis of indeterminate potential (CHIP), idiopathic cytopenias of undetermined significance (ICUS) and clonal cytopenias of undetermined significance (CCUS). *ASXL1* mutation is much more common in pre-malignant conditions than in apparent malignancies, and this is one of the most common mutations in low-risk myelodysplastic syndromes [1] (MDS) and ICUS [2] patients, in which most patients in the latter cohort harbored no mutations other than *ASXL1*. Furthermore, *ASXL1* mutation is also involved in patients with aplastic anemia [3], which suggests that *ASXL1* mutation is associated not only with myeloid transformation but also wuth bone marrow stagnation and/or failure.

Although several studies have so far elucidated the molecular mechanisms how mutant *ASXL1* contributes to myelodysplasia and promotes development of myeloid leukemia in combination with other gene alterations [4, 5], few studies have approarched what causes the impared hematopoiesis in *ASXL1*-mutated hematopoietic stem cells. And there were few models mimicking heterozygous truncated mutants, which are commonly found in patient samples, so a precise model was urgently needed.

So we generated *Asxl1* mutant locus knock-in (KI) mice, and investigated the biological effects of the mutant. Intriguingly, heterozygous mutant mice developed phenotypes recapitulating human low-risk MDS, and the impared hematopoiesis shown in the KI mice was explained by cellular senescence induced by derepression of *p16Ink4a* due to perturbation of Bmi1-driven H2AK119ub1 histone modification [6]. On the other hand, the impact of tri-methylation of H3K27 and resultant upregulation of posterior *Hoxa* genes were limited compared with previous *ASXL1* mutant or knock-out mice models. This might be in part due to the difference in expression level of mutant *ASXL1*, since previous models are bone marrow transplantation model with overexpressed *ASXL1* mutant or a transgenic mouse. Overall, in contrast to the previous models, our mice with physiolosical expression of *Asxl1* mutant showed features of pre-malignant, bone marrow failure-like phenotype. This is compatible with a previous study indicating high expression of *p16Ink4a* in low-risk MDS patient samples compared with those of high-risk MDS and AML patients [7]. The association between *ASXL1* and cellular senescence has also been pointed out in *Asxl1* null embryonic fibroblasts [8], and was further confirmed in a heterozygous KI cells this time *in vivo*.

Then, our next question is “How does *ASXL1* mutation contribute to the development of myeloid leukemia from pre-malignant condition?” There are several hypotheses for this proposition. In consideration of the phenotypes of *Asxl1* mutation KI mice, does the myeloid transformation occur only with additional mutations? Are the *ASXL1*-mutated cells vulnerable to DNA injury? Or do the senescent cells themselves exert oncogeneic potential? In human hematological disorders, the hotspot of *ASXL1* mutation is G646WfsX12, in which additional guanine is inserted into the genomic region of GGGGGGGG cassette as a frameshift mutation in exon 12. This somatic mutation is mostly found in elderly patients and healthy individuals with CHIP, and those people may be prone to acquire other mutations due to life-long accumulation of reactive oxygen species and/or other environmental stress. Several studies elucidated that senescent cells secrete inflammatory cytokines and chemokines called senescence-associated secretory phenotype, and promote tumorigenesis in solid tumor [9], and it is suggested that similar paracrine and endocrine mechanisms may exist in myeloid transformation of *ASXL1* mutant cells. Another report indicates that those cancer cells experienced chemotherapy-induced cellular senescence could change stemness-related properties of malignant cells and obtain aggressiveness [10]. These findings suggest that senescent cells, which were previously thought to act as a tumor suppressor, also harbor tumor-promoting capacity, and their pharmacologic inhibition may yield a novel therapeutic strategy in myeloid neoplasms. And *Asxl1* mutant KI mouse would be a good model to clarify the mechanism how pre-malignant cells transform into myeloid leukemia.

In summary, our findings in *Asxl1* mutant KI mice highlight a novel perspective regarding the importance of cellular senescence in the pathogensis of CHIP, ICUS, CCUS and low-risk MDS. This work also suggests possibility for discoveries of novel mechanisms in myeloid transformation. We anticipate that this model provides an ideal platform for both unveiling the molecular basis in *ASXL1* mutation-associated impaired hematopoiesis and developing novel therapies for these patients.
